# Effects of Tributyrin Supplementation on Liver Fat Deposition, Lipid Levels and Lipid Metabolism-Related Gene Expression in Broiler Chickens

**DOI:** 10.3390/genes13122219

**Published:** 2022-11-26

**Authors:** Tiantian Gu, Mingcai Duan, Jinyu Liu, Li Chen, Yong Tian, Wenwu Xu, Tao Zeng, Lizhi Lu

**Affiliations:** State Key Laboratory for Managing Biotic and Chemical Threats to the Quality and Safety of Agro-Products, Institute of Animal Science & Veterinary, Zhejiang Academy of Agricultural Sciences, Hangzhou 310021, China

**Keywords:** tributyrin, fat deposition, lipid metabolism, gene expression, broiler chicken

## Abstract

The objective of this study was to investigate the effects of tributyrin supplementation on liver fat metabolism in broiler chickens. Two hundred and forty broilers were randomly allocated into two experimental groups (6 replicates per treatment; 20 chickens in each replicate): the control group (CN), which received a basal diet, and the tributyrin group (TB), which received a basal diet supplemented with 1 g/kg of tributyrin. The experimental period lasted 37 days. The results showed that in the liver, broilers supplemented with tributyrin had higher content of high-density lipoprotein cholesterol (HDL-C) (*p* < 0.05). Liver hepatic lipase (HL), lipoprotein lipase (LPL) and total lipid (TL) activity were significantly lower than in the TB group than that in the NC group. Meanwhile, the diet supplemented with tributyrin had more lipid droplets than the NC group, whereas the TB and NC groups showed no histological abnormalities in the liver. Furthermore, the mRNA expression levels of peroxisome proliferators-activated receptor α (*PPARα*), proliferators-activated receptor γ (*PPARγ*), fatty acid synthase (*FAS*), *LPL* and adipose triglyceride lipase (*ATGL*) in the liver were significantly upregulated in the TB group (*p* < 0.05), while those of the long-chain acyl-CoA-synthetase 1 (*ACSL1*) mRNA between the TB group and the NC group were not different (*p* > 0.05). These findings indicated that the diet supplemented with tributyrin could increase fat deposition appropriately by promoting fat synthesis without causing liver tissue damage, which demonstrated that tributyrin can be considered a valid feed additive for broiler chickens.

## 1. Introduction

Chickens are important components of human food, and appropriate fat deposition in chicken meat contributes significantly to its quality attributes, such as juiciness, flavor, taste and other organoleptic properties. However, excessive fat deposition in broiler chickens will not only induce broiler ascites syndrome, sudden death and other metabolic diseases, but could also lead to adverse effects in the consumer’s health [1]. Therefore, it is imperative to find suitable additives for fat deposition.

Tributyrin (TB) is a triglyceride containing three butyrate moieties, and one molecule of tributyrin releases three molecules of butyrate directly in the small intestine, which could be rapidly adsorbed [2,3]. Supplementation of tributyrin showed positive effects on growth performance and gut health in pig and rat [4,5,6]. In pig, a basal diet supplemented with tributyrin significantly increased production traits and nutrient metabolism to regulate lipid metabolism [4]. In rat, treatment with tributyrin attenuated diet-induced obesity and associated insulin resistance [6]. Moreover, regarding beneficial effects, tributyrin administration can also attenuate lipopolysaccharide-induced liver injury through gut regulation [7,8]. Therefore, these findings indicated that tributyrin could modulate liver lipid metabolism. However, there are no other studies investigating the effects of dietary tributyrin on lipid metabolites in healthy chickens.

In birds, the liver is the main site of lipogenesis [9], contributing 80 to 85% of the fatty acids stored in adipose tissue [10]. As a result, most of the endogenous body lipids are of hepatic origin, and the development of adipose tissue depends on the availability of plasma triglycerides that are hydrolyzed prior to their utilization by adipocytes [11,12,13]. Liver fatty acid metabolism, as the most important lipid metabolic pathway, is widely involved in fat deposition [14,15]. These results prompted studies on gene expression in the liver, especially those genes involved in lipogenesis and lipolysis [16,17,18], including peroxisome proliferators-activated receptor α (*PPARα*), peroxisome proliferators-activated receptor γ (*PPARγ*), fatty acid synthase (*FAS*), adipose triglyceride lipase (*ATGL*) and lipoprotein lipase (*LPL*). However, little is known about the effect of tributyrin on hepatic gene expression in poultry, especially in broiler chickens.

Broiler chickens have a high propensity for lipid biosynthesis. It is reported that total body lipids of growing chickens double every 5.5 days, and they reach the maximum rate of hepatic fatty acid synthesis at 7 weeks of age [19]. The aim of the present study was to explore the effect of tributyrin on hepatic lipid metabolism and lipid metabolism-related gene expression in broiler chickens, which might help to identify the underlying mechanism of tributyrin in modulating fat deposition in liver tissue.

## 2. Materials and Methods

### 2.1. Ethics Statement

Animals used in this study were raised and slaughtered in accordance with the national standard of Laboratory animal Guideline for ethical review of animal welfare (GB/T 35892-2018), issued by General Administration of Quality Supervision, Inspection and Quarantine of the people’s Republic of China and Standardization Administration of the People’s Republic of China. All experiment procedures were approved by the Institute of Animal Husbandry and Veterinary Science, Zhejiang Academy of Agricultural Sciences (Hangzhou, China).

### 2.2. Animals and Experimental Model

Two hundred and forty 26-day-old Hexi dwarf female broilers were randomly allotted into two experimental groups with similar conditions to those under which commercial farm animals were kept before the first day of the trial. After one week of adaptation, the control group (NC) received a basal diet, while the TB group received the same basal diet supplemented with 1 g/kg of dietary tributyrin [20] (Jinfulai Technology Development Co., Ltd., Harbin, China). All groups consisted of 6 replicates per treatment and 20 chickens in each replicate. The dietary nutrient levels were based on National Research Council recommended nutrient requirements for broiler chickens (Table 1). The experimental period lasted 37 days. After the experimental period, six broilers in each group were randomly selected for blood and liver sample collection.

### 2.3. Sample Collection

After 37 days, blood samples from the jugular vein of six broiler chickens per treatment (1 broiler per replicate) were collected into pro-coagulation tubes and maintained for 2 h at room temperature. All samples were centrifuged at 3000 rpm for 10 min at 4 °C. Serum was removed and the aliquots were stored at −20 °C for further analysis. Broilers were euthanized with an ear intravenous injection of sodium pentobarbital (200 mg/kg BW). The liver tissue samples were collected and immediately snap-frozen in liquid nitrogen and fixed in 4% paraformaldehyde for RNA and histological analysis, respectively.

### 2.4. Biochemical Analysis 

Liver total cholesterol (TC), triglycerides (TG), high-density lipoproteincholesterol (HDL-C) and low-density lipoproteincholesterol (LDL-C) were measured using commercially available kits (Jiancheng Biotechnology Inc., Nanjing, China), following the manufacturers’ instructions. Hepatic lipase (HL), LPL and total lipid (TL) activities were determined using liver tissue HL, LPL and TL kits (Jiancheng Biotechnology Inc., Nanjing, China), following the manufacturers’ instructions. 

### 2.5. Histological Observation

Liver tissues fixed in 4% paraformaldehyde were subjected to a standard hematoxylin and eosin (H&E) staining and then cut into 10 μm thin layers. The obtained slices were subjected to a dry-wash cycle (60 min); after that, the dried slices were rinsed in 60% isopropanol and then stained with Oil Red O solutions (10 min). After differentiation in 60% isopropanol, distilled water was used to wash twice, then restained with Mayer’s hematoxylin (2 min), washed twice with distilled water (10 min), dried and embedded with the aqueous medium. The slices were observed using a Nikon E100 microscope, and pictures were taken at a magnification of 400×.

### 2.6. Gene Expression Analysis

Total RNA was isolated from the liver samples using TRIzol reagent (TAKARA, Dalian, China), and then the single-stranded cDNA was synthesized. All cDNA samples were stored at −20 °C until used. A 20 μL reaction mixture contained 10 μL of 2× Power SYBR^®^ Green Master Mix (Applied Biosystems, Waltham, MA, USA), 0.5 μL of each primer, 1 μL of template cDNA, and 8 μL of double-distilled water (ddH_2_O). The amplification reaction consisted of 95 °C for 30 s, then 40 cycles at 95 °C for 15 s and 63 °C for 25 s. Glyceraldehyde 3-phosphatedehydrogenase (GAPDH) was used as the housekeeping gene, and the normalized target gene expression level in the sample was calculated using the formula 2^−ΔΔCt^. The primers were designed using Primer Premier 5.0 software and synthesized by Sangon Biotech (Shanghai, China), and they are listed in Table 2.

### 2.7. Statistical Analysis

Data are presented as the mean ± standard error (SE) and processed by the statistical package for the SPSS 25.0 software (Chicago, IL, USA). *t*-test analysis was used to assess the differences between groups. Differences were considered statistically significant at *p* < 0.05.

## 3. Results

### 3.1. Effect of Tributyrin on Lipid Levels in Liver Tissues

To determine whether dietary addition of TB could affect fat deposition in the liver, the contents of TC, TG, HDL-C and LDL-C were observed. As indicated in Table 3, TB treatment significantly increased the content of HDL-C (*p* < 0.05), while no difference between the control and TB groups in the liver content of TC, TG and LDL-C (*p* > 0.05) was observed. On the other hand, broilers fed the diet supplemented with TB had significantly decreased values of the liver TL, HL and LPL activity.

### 3.2. Effect of Tributyrin on Enzymatic Activity in Liver Tissues

As shown in Table 4, the hepatic activity of TL in the TB supplementation group was significantly lower than in the control group (*p* < 0.05). Meanwhile, the activity of HL and LPL in the liver were also significantly lower in the TB supplementation group (*p* < 0.05). 

### 3.3. Histological Observations of Liver Tissues

**Figure 1:**
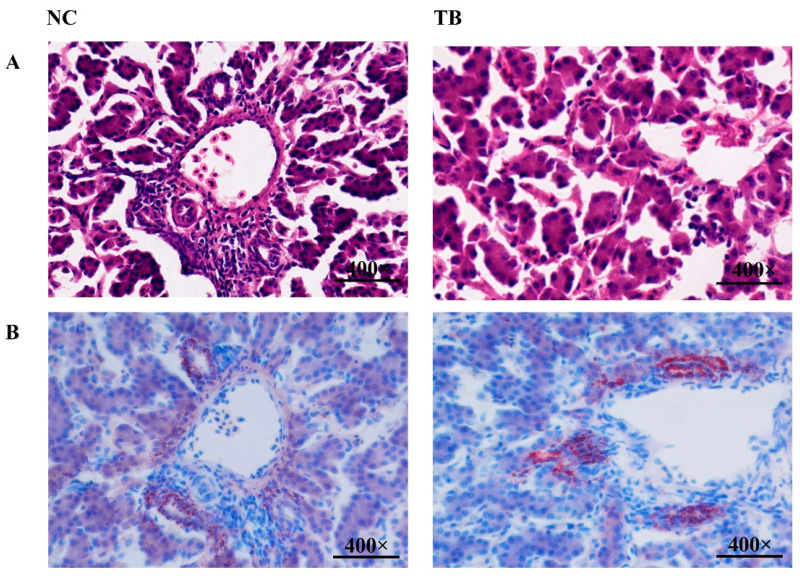
TB increased fat deposition in the liver tissue. (**A**) H&E staining of liver tissue. (**B**) Oil Red O staining.

To confirm the changes in liver tissue after TB supplementation, histological analysis was performed, and the NC and TB groups showed no histological abnormalities in the liver (Figure 1A). To further confirm the distribution and abundance of lipid droplets, the liver tissue was subjected to Oil Red O staining. It was found that the TB group had more lipid droplets than the NC group (Figure 1B).

### 3.4. Effect of Tributyrin on Liver mRNA Expression of Lipogenesis and Lipolysis-Related Genes 

**Figure 2:**
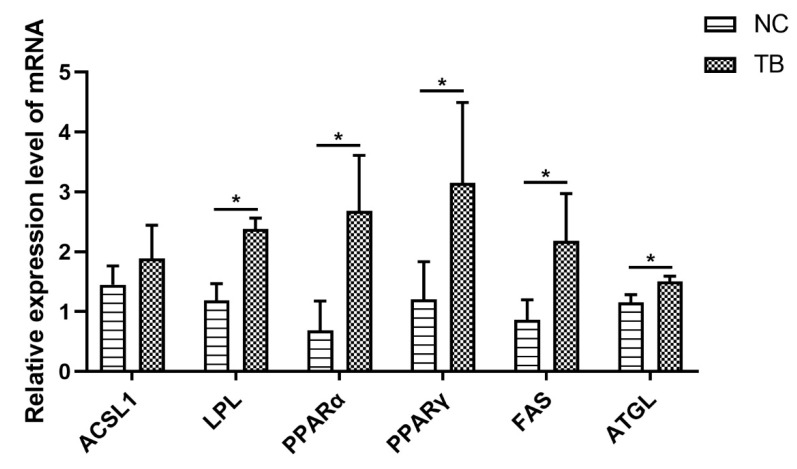
TB improved the lipid metabolism in the liver. Values are expressed as the mean ± SE (*n* = 4). * indicates significant difference (*p* < 0.05).

To further determine the underlying mechanisms of tributyrin involved in liver lipid deposition, the expression levels of genes that are related to lipogenesis and lipolysis were measured (Figure 2). Among lipid uptake genes, the mRNA expression levels of *LPL* in the TB group were significantly higher than that in the control group (*p* < 0.05), while there was no difference between the control and TB groups in *ACSL1* mRNA expression (*p* > 0.05). The key lipogenesis genes, including *PPARα*, *PPARγ* and *FAS*, were also compared in the two groups. It was found that in comparison to the control group, TB treatment significantly increased *PPARα*, *PPARγ* and *FAS* mRNA expression levels (*p* < 0.05). Finally, TB was found to increase the expression of the *ATGL* gene related to lipolysis in the liver (*p* < 0.05).

## 4. Discussion

With its long history as a delicious food source, chicken has proven highly popular with consumers. Meanwhile, appropriate fat deposition in broiler chickens will improve the flavor of meat quality, which makes the meat more delicious [21]. Glyceryl butyrate has beneficially improved the growth performance and carcass yield in broiler chickens [22,23,24]. Moreover, TB contains the butyrate moieties and could be rapidly adsorbed by releasing the butyrate directly in the small intestine [2,3]. In birds, lipogenesis takes place primarily in the liver, whereas adipocyte serves as the storage site for triglycerides [9]. Despite the wealth of information indicating that tributyrin alters lipid metabolism in animals [4,6], little is known about the effect of tributyrin on lipid metabolism and hepatic gene expression in poultry, especially in broiler chickens.

The HDL-C can transport the free cholesterol accumulated in peripheral tissues and the lipoproteins in circulation to the liver cells, accelerating the removal of cholesterol and thus playing an important role in minimizing atherosclerosis [25,26]. In the present study, broiler chickens fed with TB-containing diets showed increased content of HDL-C in liver tissue, while no statistically significant differences were observed for TC, TG and LDL-C content. These results indicated that TB could accelerate the accumulation of HDL cholesterol in circulation.

The lipid mobilization includes lipogenesis and lipolysis, which are in a dynamic balance under normal conditions [27]. The HL, as an important lipid metabolic enzyme produced primarily by the liver, mainly modulates the reaction responsible for transferring cholesterol from the peripheral tissue to the liver [28]. The LPL is synthesized in parenchymal cells in fat, myocardium, skeletal muscle and breast tissues, which has functional similarities with HL. HL has been found to catalyze and break the TG into fatty acids and monoglycerides for use in aerobic metabolism or for fat storage. The liver takes up the unesterified cholesterol accumulated in HDL with the help of HL. This could prevent excess cholesterol accumulation in the liver’s peripheral tissues [29,30]. This study demonstrated a pronounced increase in HL, LPL and TL activity after the administration of TB in broiler chickens, which resulted in increased lipid synthesis and accelerated lipolysis [28,31]. Histological analysis and Oil Red O staining further demonstrated that TB dietary supplementation accelerated liver fat deposition with no adverse effects on hepatic functionality. We speculated that TB might positively affect liver lipid regulation based on the findings mentioned above.

Fat accumulation is a complex process characterized by many gene expression changes controlling lipogenesis and lipolysis [1]. LPL, a classical lipid metabolic enzyme, is involved in liver fatty acid metabolism [32], and ACSL1 is also involved in the regulation of lipid metabolism uptake [33]. Our study showed that the mRNA expression level of LPL in the TB group was higher than in the NC group, while the *ACSL1* mRNA level showed no difference between the TB and NC groups. The result suggested that TB may improve lipid uptake of chicken broilers by regulating the *LPL* level. The PPARs are a superfamily of nuclear receptors that play a significant role in adipocyte cell differentiation and intra- and extracellular transportation of fatty acid [34,35]. Both PPARα and PPARγ mainly influence fatty acid metabolism and modulate the lipid accumulation via increased expression of *LPL* [36]. In the present study, we found that TB treatment significantly upregulated the *LPL*, *PPARα* and *PPARγ* mRNA levels, showing similarities with a previous study where oral tributyrin increased the hepatic PPARα and PPARγ gene expression to attenuate LPS-induced lipid metabolism abnormalities [37]. Based on these results, supplementation with TB could play a positive role in liver lipogenesis in chicken broilers. Moreover, ATGL is the key lipase involved in the lipolysis process, and these enzymes hydrolyze the triacylglycerols to monoacylglycerols and other lipids in various tissues [38,39]. The TB group had a higher *ATGL* mRNA expression than the NC group, which indicated that TB might improve the liver lipolysis of chicken broilers. These data suggest that TB plays a more relevant role in balancing lipogenesis and lipolysis.

## 5. Conclusions

The present study showed that supplementation with tributyrin significantly elevated HDL-C content and decreased the HL, LPL and TL activity. Broiler chickens treated with tributyrin promoted fat deposition without negative effects on liver morphology. Moreover, the *PPARα*, *PPARγ*, *FAS*, *LPL* and *ATGL* mRNA levels were significantly upregulated. All these data suggested that tributyrin could promote liver fat deposition without resulting in excessive accumulation. For these reasons, it would be interesting to evaluate and investigate the meat quality in detail.

## Figures and Tables

**Table 1:** The composition and nutrient levels of the experimental diets.

Items	26~40 d	41~62 d
Ingredient (%)		
Wheat	77.96	81.51
Soybean meal	5.65	0
Sunflower meal	3.5	4.5
Peanut meal	4	4.25
Corn gluten meal	1.56	1.28
Feather meal	0	1
Lard	3.14	3.46
Calcium bicarbonate	0.63	0.47
Stone powder	1.36	1.33
Premix ^1^	2.2	2.2
Nutrient composition, calculated		
Nitrogen-corrected apparent metabolizable energy (MJ/kg)	12.76	12.97
Crude protein (%)	17.50	16.50
Crude fat (%)	4.76	5.17
Calcium (%)	0.8	0.75
Phosphorus (%)	0.3	0.28
Lysine (%)	0.90	0.85
Methionine (%)	0.45	0.37

^1^ Premix supplied the following per kilogram of diet: NaHCO_3_ 90 g; NaCl 90 g; vitaminA 10,000 IU; vitaminD 33,000 IU; vitaminE 30 mg; vitaminK 31.3 mg; vitaminB 120.013 mg; thiamine 2.2 mg; riboflavin 8 mg; nicotinamide 40 mg; choline chloride 600 mg; calcium pantothenate 10 mg; pyridoxine·HCl 4 mg; biotin 0.04 mg; folic acid 1 mg; Fe 80 mg; Cu 7.5 mg; Mn 110 mg; Zn 65 mg; I 1.1 mg; Se 0.3 mg. Moreover, 26 to 40 days includes methionine 90 g; threonine 70 g; lysinesulphate 361 g, while 41 to 62 days includes methionine 68 g; threonine 89 g; lysinesulphate 406 g.

**Table 2:** Primers used in the study.

Gene	Genbank Accession	Primer Sequences (5′→3′)	Size (bp)	Annealing (°C)
β-actin	NM_205518.1	CTGAACCCCAAAGCCAACAGA	120	60
		AGTGGTACGACCAGAGGCATACA		
LPL	NM_205282.2	CAGTGTCTGCTGCTTACACGAA	101	60
		CAAGTGGACATTGTTGAGAGGGTAA		
ACSL1	XM_040698931.1	CGGACAGAGCAGAGTATGTG	74	60
		GCCTACGTACTGGCTGTGA		
PPARγ	NM_001001460.1	CATGCATCACCACTGCAGGAA	83	60
		ACTGCCTCCACAGAGCGAAA		
PPARα	NM_001001464.1	GGAGTACATGCTTGTGAAGGTTG	148	60
		CTGAAAGGCACTTCTGAAAACGACA		
FAS	NM_205155.3	CAAGCCTGGAGATGTGGAGTAT	154	60
		CTCTGGATGACCCATGTTTGAC		
ATGL	EU240627.2	CTGACAACTTGCCACGATATGAG	149	60
		GAGGTTGCGAAGGTTGAATTGGA		

**Table 3:** Effect of TB on liver lipid parameters in broiler chickens.

	NC	TB	*p*-Value
TC (mmol/L)	0.0645 ± 0.0079	0.0578 ± 0.0059	0.129
TG (mmol/L)	0.1248 ± 0.0187	0.1073 ± 0.0135	0.095
HDL-C (mmol/L)	0.0049 ± 0.0008 ^b^	0.0071 ± 0.0005 ^a^	<0.001
LDL-C (mmol/L)	0.0532 ± 0.0058	0.0480 ± 0.0057	0.149

TC, total cholesterol; TG, triglycerides; HDL-C, high-density lipoproteincholesterol; LDL-C, low-density lipoproteincholesterol. Data are presented as means ± SE (*n* = 6). Different superscripts indicate significant differences between groups (^a^, ^b^: *p* ≤ 0.05).

**Table 4:** Effect of TB on liver lipid parameters in broiler chickens.

	NC	TB	*p*-Value
TL (U/mg.prot)	9.14 ± 0.73 ^a^	6.30 ± 0.96 ^b^	<0.001
HL (U/mg.prot)	1.78 ± 0.32 ^a^	1.23 ± 0.14 ^b^	0.003
LPL (U/mg.prot)	7.36 ± 0.47 ^a^	5.08 ± 0.84 ^b^	<0.001

TL, totallipase; HL, hepatic lipase; LPL, lipoproteinlipase. Data are presented as means ± SE (*n* = 6). Different superscripts indicate significant differences between groups (^a^, ^b^: *p* ≤ 0.05).

## Data Availability

Not applicable.
